# Supplementary material for: Simulation-based Reconstructed Diffusion unveils the effect of aging on protein diffusion in Escherichia coli
Source: PLoS Comput Biol. 2023 Sep 11;19(9):e1011093. doi: 10.1371/journal.pcbi.1011093 (PMC10513214; doi:10.1371/journal.pcbi.1011093)
Supplement: S1 Text — (PDF) [file pcbi.1011093.s001.pdf]

Supporting text – A mathematical solution to confined diffusion

We previously showed through simulations that the measured diffusion coefficient is underestimated when particles move by random motion in a confined environment. The deviation from the real diffusion coefficient drastically increases when the diffusion coefficient gets higher, and the analyzed molecules are in a region closer to the boundary of the confinement [14] (Supporting Fig. 1A).

We now developed a mathematical model to solve this limitation. The simplest approach to modeling of diffusion is through Ficks laws, which in his second law led to the so-called diffusion equation (Eq. 1)

$$\frac{\partial\rho(x,t)}{\partial t} = D\Delta\rho(x,t) \tag{1}$$

In the context of random motion, say a random walk of a particle, ρ denotes a probability density (or distribution) and $P(R) = \int_R \rho \, \mathrm{d}x$ would be the probability of finding the particle within a region R.

Here we solve analytically the one-dimensional diffusion equation with reflecting boundaries. For simplicity, we interpret $\rho(x, t)$ as the probability density for finding a particle at position x at time t. Assuming that at $t = 0$ the particle is located at $x = 0$, and that the probability density $\rho(x, t) = 0$ as x approaches infinite for any finite time, a solution to equation 1 is then given by equation 2:

$$\rho(x,t) = \frac{1}{\sqrt{4\pi Dt}} \exp\left(-\frac{x^2}{4Dt}\right) \tag{2}$$

In a heuristic way, equation 2 provides a measure of the likelihood of finding a particle at position x after time t provided that the medium where it moves is infinite.

Let us now consider the diffusion of a particle within a bounded domain, for simplicity from $-L/2$ to $+L/2$, where L is the length of the domain. We assume that the particle is located at $x = 0$ at $t = 0$, and that it is reflected back to the interior of the interval once it reaches the boundary (Supporting Fig. 1B). The analytical solution for this case is given by equation 3 (see Supporting Information – Diffusion on a closed interval):

$$\rho_L(x,t) = \frac{1}{L} + \frac{2}{L}\sum_{n=1}^{\infty} \cos\left(\frac{2\pi nx}{L}\right) \exp\left(-\frac{4\pi^2 n^2 Dt}{L^2}\right) \tag{3}$$

Relying on an analytical solution of the diffusion equation in higher dimensions and for more complicated geometries is not convenient. Equation 2 is valid for diffusion in unbounded domains. We can approximate the solution of equation 3 using a "folding approach", by accounting for the bounces a particle makes when hitting the boundaries, assuming no loss of energy in the process, and adding them up (Supporting Fig. 1C).

In this way, an approximation of equation 3 is given by equation 4:

$$\tilde{\rho}(x,t) = \rho(x,t) + \rho(L-x,t) + \rho(L+x,t) + \rho(2L-x,t) + \rho(2L+x,t) + \cdots \quad (4)$$

where the term $\rho(kL-x) + \rho(kL+x)$ corresponds to the density for the particle being at position x at time t after k bounces. When substituting equation 2 in equation 4 we get:

$$\tilde{\rho}(x,t) = \frac{1}{\sqrt{4\pi Dt}}\left[\exp\left(-\frac{x^2}{4Dt}\right) + \sum_{k=1}^{N}\left(\exp\left(-\frac{(kL-x)^2}{4Dt}\right) + \exp\left(-\frac{(kL+x)^2}{4Dt}\right)\right)\right] \quad (5)$$

where N denotes the maximum number of bounces (Supporting Fig. 1C).

One can take a similar approach in higher dimensions. For example, we consider the diffusion in a rectangular plate of sides A (horizontal) and B (vertical), and here we assume that the motion of the particle on each coordinate (x, y) is independent of each other. The analytical solution for the diffusion equation then becomes:

$$\rho(x,y,t) = \rho_A(x,t)\rho_B(x,t) \quad (6)$$

where ρ_A and ρ_B are as in equation 3.

We can however take another approach, as we did for the interval in one dimension (equation 4, 5). We denote by $p(t) = (x(t), y(t))$ the position of a particle in the rectangular plate at time t. As above, we then compute the density $\rho(x, y, t)$ by adding up all the densities corresponding to trajectories in the rectangular plate that take the particle from the initial position p_0 to some final position p_f after a number of bounces (Supporting fig. 2A, 2B). What we describe is in fact an example of a mathematical billiard.

To use these ideas to estimate the diffusion coefficient inside a cell, we approximate the geometry of the cell by a planar sphero-cylinder, also known as a Bunimovich stadium (Supporting Fig. 1D). In this setting, 0-bounce and 1-bounce trajectories can be easily computed. Densities corresponding to trajectories that bounce on the straight (top and bottom) sides of the sphero-cylinder are computed as aforementioned. Those corresponding to bounces on the circular sides can be computed by solving the system of equations:

$$\begin{cases} \dfrac{A_0 x_c + B_0 y_c}{\sqrt{A_0^2 + B_0^2}} = \dfrac{A_f x_c + B_f y_c}{\sqrt{A_f^2 + B_f^2}} \\ x_c^2 + y_c^2 = R^2 \end{cases} \quad (7)$$

where $A_0 = y_c - y_0$, $B_0 = x_c - x_0$, $A_f = y_c - y_f$, $B_f = x_c - x_f$, and (x_0, y_0), (x_c, y_c), (x_f, y_f) represent the starting point, a bouncing point on the circular section of the cell boundary and the end point, respectively (Supporting Fig. 1D).

Since equation 7 cannot be solved analytically, we developed a script (see Supporting Information – algorithm 1) to compute the 0-bounce and 1-bounce trajectories of any diffusing particle, provided that the start and end positions are known. We then used this algorithm together with SMdM to calculate the diffusion coefficient of two-dimensional diffusion simulations in a billiard, generated with Smoldyn (Supporting Fig. 1E). SMdM utilizes the equation $\rho(x, y, t) = \rho(x, t)\rho(y, t)$, where $\rho(x, t)$ and $\rho(y, t)$ are as in equation 2. The displacement map is obtained by using the spatiotemporal information of a particle diffusing from a start to an end position in a fixed period of time, and fitting the information into the SMdM equation (see Materials and Methods – SMdM analysis, Eq. 11) using D as fitting parameter. Smoldyn allows simulation of the motion of particles using a predefined diffusion coefficient and time resolution, within a simulation compartment. With our mathematical model we obtain a set of displacements for every diffusing particle. Such a set is composed of the 0-bounce trajectory and of all the possible 1-bounce trajectories. By adding up all the trajectories and calculating their combined density as in equation 6, we obtain a final diffusion coefficient for every pixel that is a good approximation of the input diffusion coefficient used for the simulations.

Applying the mathematical approach to solve the diffusion equation near the boundaries in confined environments has three shortcomings. (i) The approximation (equation 5) is valid for the given boundary conditions, but not for e.g. non-continuous, non-convex surfaces. An example of a surface where our model would have failed is the Penrose unilluminable room (Supporting Fig. 3A), or the matrix of mitochondria. Some regions of these surfaces are inaccessible by rays that start from particular locations, regardless of the number of bounces. However, for a particle freely diffusing in any compartment, it would be possible to reach any location, leading to the emergence of starting and final points that cannot be connected by reflecting rays. (ii) The model cannot be easily extended from the two-dimensional billiard to the three-dimensional spherocylinder. Given a start and end point in two dimensions, it is always possible to find a reflection point on a circle; on the other hand, given a start and an end point in three dimensions, there will be an infinite number of reflection points on a sphere. Therefore our model implies that the motion of particles only occurs in two dimensions (x,y coordinates of the diffusing particles), while in reality (in cells) particles also diffuse along the z-axis. When simulating diffusion in a three-dimensional spherocylinder and analyzing it with our mathematical model (equation 5), we observed an underestimation of the diffusion coefficient throughout most of the cell, and an overestimation of it close to the boundary (Supporting Fig. 3C). The underestimation is due to the motion along the z-axis, which is not accounted for in our model, leading to a measured step length shorter than the actual one (Supporting Fig. 3B, left). The overestimation is likely due to particles bouncing against the boundary at z coordinates where the spherocylinder (x,y) section has a smaller perimeter (Supporting Fig. 3B, right). (iii) Finally, when approximating the solution in a bounded domain one must compute all trajectories that lead from the initial position p_0 to the final position p_f after $0, 1, 2, \ldots, N$ bounces. Computing all these trajectories analytically is cumbersome, and therefore we limited our analysis to the 1-bounce case. This can be limiting in the case of fast diffusion in small compartments, *i.e.* when the square root of the mean squared displacement is much larger than the size of the compartment, or when the acquisition time is very long. In these cases a particle could bounce against the surface multiple times over the acquisition period.
