# Supplementary material for: Simulation-based Reconstructed Diffusion unveils the effect of aging on protein diffusion in Escherichia coli
Source: PLoS Comput Biol. 2023 Sep 11;19(9):e1011093. doi: 10.1371/journal.pcbi.1011093 (PMC10513214; doi:10.1371/journal.pcbi.1011093)
Supplement: S2 Text — (PDF) [file pcbi.1011093.s002.pdf]

Supporting text - Diffusion on a closed interval

Let us consider the diffusion problem

$$\frac{\partial p(x,t)}{\partial t} = D\frac{\partial^2 p(x,t)}{\partial x^2}, \tag{1}$$

where $a \le x \le b$ and $x_0 \in [a,b]$, $p(x,t)$ stands for the probability of finding a particle in position x at time t, and D is the diffusion constant of the medium. In other words, we are considering a diffusion problem in the closed interval $[a,b]$ with initial condition $p(x,0) = \delta(x - x_0)$, with $\delta(\cdot)$ denoting the Dirac-delta. Furthermore, we assume the boundary conditions

$$\left.\frac{\partial p(x,t)}{\partial x}\right|_{x=a} = \left.\frac{\partial p(x,t)}{\partial x}\right|_{x=b} = 0, \tag{2}$$

accounting for the "rigid" reflection at the boundaries of the interval. These are generally known as Neumann boundary conditions.

To simplify the computations, we translate the previous setting into a diffusion problem defined on the closed interval $[-\ell/2, \ell/2]$. This is done by defining $y = \alpha x + \beta$ with $\alpha = \frac{b-a}{\ell}$ and $\beta = \frac{a+b}{2}$. Denoting by $\bar{p}$ the re-scaled probability function, we now have the diffusion problem

$$\frac{\partial \bar{p}(y,t)}{\partial t} = \bar{D}\frac{\partial^2 \bar{p}(y,t)}{\partial y^2}, \qquad \bar{p}(y,0) = \delta(y - y_0), \qquad \left.\frac{\partial \bar{p}(y,t)}{\partial y}\right|_{y=\pm\frac{\ell}{2}} = 0. \tag{3}$$

To find the solution to the diffusion equation (10) we propose the ansatz

$$\bar{p}(y,t) = \bar{\phi}(y)e^{-\gamma t}. \tag{4}$$

Substituting (11) in (10) we get

$$\frac{\partial^2 \bar{\phi}}{\partial y^2}(y) + \omega^2 \bar{\phi}(y) = 0, \tag{5}$$

where $\omega^2 = \frac{\gamma}{D}$.

The eigenvalue problem given by (12) is that of an harmonic oscillator. This tells us that its solution is given by a linear combination of the eigenfunctions

$$\begin{aligned} \bar{\phi}_n^{\mathrm{e}} &= A_n \cos(\omega_n^{\mathrm{e}} y) \\ \bar{\phi}_n^{\mathrm{o}} &= B_n \sin(\omega_n^{\mathrm{o}} y), \end{aligned} \tag{6}$$

corresponding to the even and odd harmonics, respectively. The boundary conditions are translated into:

$$\begin{aligned} \frac{\partial \bar{\phi}_n^{\mathrm{e}}}{\partial y}(\pm\ell/2) &= -\omega_n^{\mathrm{e}} A_n \sin\left(\pm\omega_n^{\mathrm{e}}\frac{\ell}{2}\right) = 0 \\ \frac{\partial \bar{\phi}_n^{\mathrm{o}}}{\partial y}(\pm\ell/2) &= \omega_n^{\mathrm{o}} B_n \cos\left(\pm\omega_n^{\mathrm{o}}\frac{\ell}{2}\right) = 0, \end{aligned} \tag{7}$$

which imply:

$$\begin{aligned}\omega_n^{\mathrm{e}} &= \frac{2n\pi}{\ell},\\ \omega_n^{\mathrm{o}} &= \frac{(2n+1)\pi}{\ell},\end{aligned} \tag{8}$$

respectively. Therefore, we have that

$$\bar{\phi}(y) = \sum_{n=0}^{\infty} A_n \cos\left(\frac{2n\pi}{\ell}y\right) + B_n \sin\left(\frac{(2n+1)\pi}{\ell}y\right) \tag{9}$$

On the other hand, the ansatz () must also satisfy $\int_{-\ell/2}^{\ell/2} \bar{\phi}(y)\mathrm{d}y = 1$, therefore:

$$\begin{aligned}1 &= \int_{-\ell/2}^{\ell/2} \left(\sum_{n=0}^{\infty} A_n \cos\left(\frac{2n\pi}{\ell}y\right) + B_n \sin\left(\frac{(2n+1)\pi}{\ell}y\right)\right) \mathrm{d}y\\ &= \left(\sum_{n=0}^{\infty} \frac{A_n\ell}{2n\pi} \sin\left(\frac{2n\pi}{\ell}y\right) - \frac{B_n\ell}{(2n+1)\pi} \cos\left(\frac{(2n+1)\pi}{\ell}y\right)\right)\Bigg|_{-\ell/2}^{\ell/2}\\ &= \sum_{n=0}^{\infty} \frac{A_n\ell}{n\pi} \sin(n\pi) = A_0\ell,\end{aligned} \tag{10}$$

which implies $A_0 = \frac{1}{\ell}$. The rest of the coefficients of (16) are obtained from the initial conditions as follows.

The Fourier series of $\delta(\xi)$, for $\xi \in [-\pi, \pi]$ is

$$\delta(\xi) = \frac{1}{2\pi} + \frac{1}{\pi}\sum_{n=1}^{\infty} \cos(n\xi). \tag{11}$$

By shifting and re-scaling as previously, we can write the Fourier series of $\delta(y - y_0)$, with $y_0 \in [-\ell/2, \ell/2]$, as

$$\delta(y - y_0) = \frac{1}{\ell} + \frac{2}{\ell}\sum_{n=1}^{\infty} \cos\left(\frac{2\pi n}{\ell}(y - y_0)\right). \tag{12}$$

So, since (16) is independent of time we immediately get

$$\bar{\phi}(y) = \frac{1}{\ell} + \frac{2}{\ell}\sum_{n=1}^{\infty} \cos\left(\frac{2\pi n}{\ell}(y - y_0)\right). \tag{13}$$

This leads to

$$\bar{p}(y,t) = \left(\frac{1}{\ell} + \frac{2}{\ell}\sum_{n=1}^{\infty} \cos\left(\frac{2\pi n}{\ell}(y - y_0)\right)\right) e^{-\gamma t}, \tag{14}$$

where, by definition, $\omega^2 = \frac{4\pi^2 n^2}{\ell^2}$ and therefore $\gamma = \frac{4\pi^2 n^2}{\ell^2}\bar{D}$. Returning to the original coordinate x, we finally obtain:

$$p(x,t) = \frac{1}{b-a} + \frac{2}{b-a}\sum_{n=1}^{\infty} \cos\left(\frac{2\pi n}{b-a}(x-x_0)\right) \exp\left(-\frac{4\pi^2 n^2 D}{(b-a)^2}t\right). \tag{15}$$
