# Supplementary material for: Simulation-based Reconstructed Diffusion unveils the effect of aging on protein diffusion in Escherichia coli
Source: PLoS Comput Biol. 2023 Sep 11;19(9):e1011093. doi: 10.1371/journal.pcbi.1011093 (PMC10513214; doi:10.1371/journal.pcbi.1011093)
Supplement: S1 Algorithm — We assume that the coordinate system is located at the center of the billiard. (PDF) [file pcbi.1011093.s003.pdf]

Algorithm 1 General algorithm used to estimate the diffusion coefficient from the billiard approach. We assume that the coordinate system is located at the center of the billiard.

procedure ESTIMATE $D(p_0, p_f, R, L)$

1: $d_0 = ||p_f - p_0||$

2: $d_1 = F_s(p_0, p_f, L, R) + F_c(p_0, p_f, L, R)$

3: Using $\tilde{\rho}(d,t) = \rho(d_0,t) + \rho(d_1,t)$, where $\rho(d,t) = \frac{1}{4\pi Dt}\exp\left(-\frac{d^2}{4Dt}\right)$ perform a maximum likelihood estimation using D as fitting parameter.

end procedure

function $F_s(p_0, p_f, L, R)$ # Reflection points on straight segments
  $(x_0, y_0) \leftarrow p_0$
  $(x_f, y_f) \leftarrow p_f$
  $x_c^u = \frac{(R-y_0)(x_f-x_0)}{2R-y_f-y_0} + x_0$ # x-coordinate of the upper point
  $x_c^b = \frac{(-R-y_0)(x_f-x_0)}{-2R-y_f-y_0} + x_0$ # x-coordinate of the bottom point
  if $|x_c^u| \leq L/2$ **then**
    $p_c = (x_c^u, R)$
    $d_u = ||p_c - p_0|| + ||p_c - p_f||$
  else
    $d_u = 0$
  end if
  if $|x_c^b| \leq L/2$ **then**
    $p_c = (x_c^b, -R)$
    $d_b = ||p_c - p_0|| + ||p_c - p_f||$
  else
    $d_b = 0$
  end if
  return $d_u + d_b$
end function

function $F_c(p_0, p_f, L, R)$ # Reflection points on curved segments
  $p_f \mapsto p_f - \frac{L}{2}$ # The next computation is done with the origin at $(L/2, 0)$
  $P_c = (x_c, y_c) \leftarrow$ solution from (8) # May give more than one solution. In that case P_c is an array of tuples $p_c = (x_c, y_c)$
  $d_r = \sum_{\substack{p_c \in P_c \\ x_c > 0}} ||p_c - p_0|| + ||p_c - p_f||$ # Add up all 1-bounce distances of points reflecting on the right circular segment
  $p_f \mapsto p_f + \frac{L}{2}$ # The next computation is done with the origin at $(-L/2, 0)$
  $P_c = (x_c, y_c) \leftarrow$ solution from (8) # May give more than one solution. In that case P_c is an array of tuples $p_c = (x_c, y_c)$
  $d_l = \sum_{\substack{p_c \in P_c \\ x_c < 0}} ||p_c - p_0|| + ||p_c - p_f||$ # Add up all 1-bounce distances of points reflecting on the left circular segment
  return $d_r + d_l$
end function
