# Supplementary figures and images for: Simulation-based Reconstructed Diffusion unveils the effect of aging on protein diffusion in Escherichia coli
Source: PLoS Comput Biol. 2023 Sep 11;19(9):e1011093. doi: 10.1371/journal.pcbi.1011093 (PMC10513214; doi:10.1371/journal.pcbi.1011093)

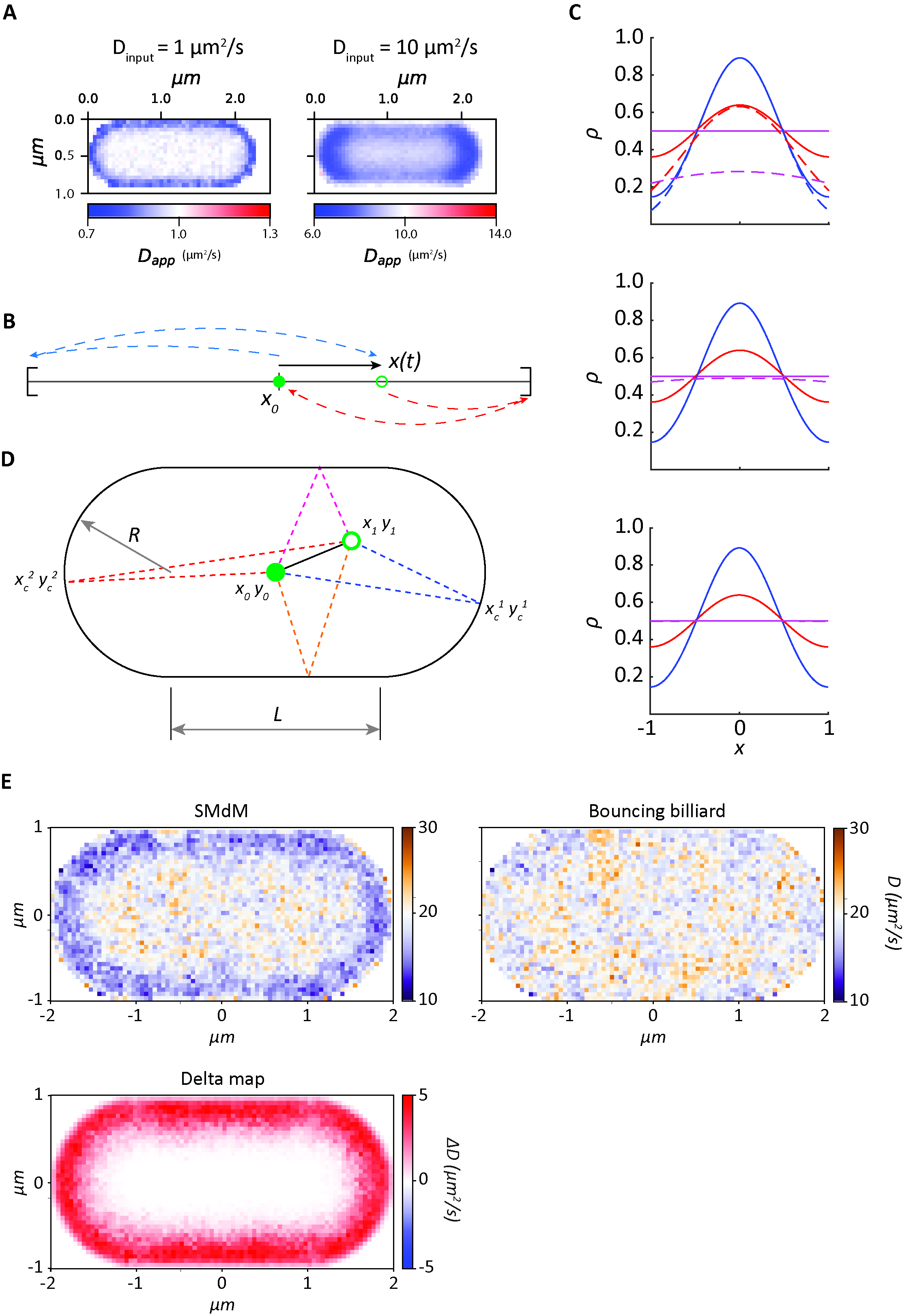

Supplement: S1 Fig — (A) Effect of confinement on diffusion. Diffusion simulations performed in a billiard at different input diffusion coefficients. The position of the particles was measured every 1.5 ms. The higher the diffusion coefficient used for the simulation, the more pronounced the confinement effect is. (B). Sketch of the (random) motion of a particle in a 1 dimensional closed interval. The point x0 stands for the initial position of the particle at t = 0. The point x(t) represents the position of the particle at time t > 0. In this scenario, and for fixed t > 0, there are several possibilities for measuring a particle’s position at x(t). The first scenario is that the particle travels to its measured position without bouncing. Another scenario is that the particle arrives at its measured position after bouncing (on the boundaries) once. In this way, there are infinitely many ways in which the particle can reach its measured position, depending on the number of bounces the particle made. However, the probability of each case is inversely proportional to the total distance traveled (Eq 3, Supporting Information–Diffusion on a closed interval). In B we show the distances traveled for 0 and 1 bounce (against each boundary). (C) Solutions of the diffusion equation on a bounded interval with length L = 2 μm, D = 2 μm2/s, and t = {0.05, 0.1, 0.5} seconds, shown in blue, red, and purple respectively. The solid lines correspond to the analytical solution [3], while the dashed curves correspond to [5]. From top to bottom we show comparisons for 0, 1, and 2 bounces. Notice that, in this example, accounting for two bounces already gives a sufficiently good approximation of the analytical solution (bottom panel). (D) A few trajectories of a billiard in the Bunimovich stadium. We show one 0-bounce (solid line) and four 1-bounce billiard trajectories (dashed lines). The bouncing points (xc1, yc1) and (xc2, yc2) are solutions of the system of equations shown under [7]. (E) Diffusion maps of [file pcbi.1011093.s004.tif]

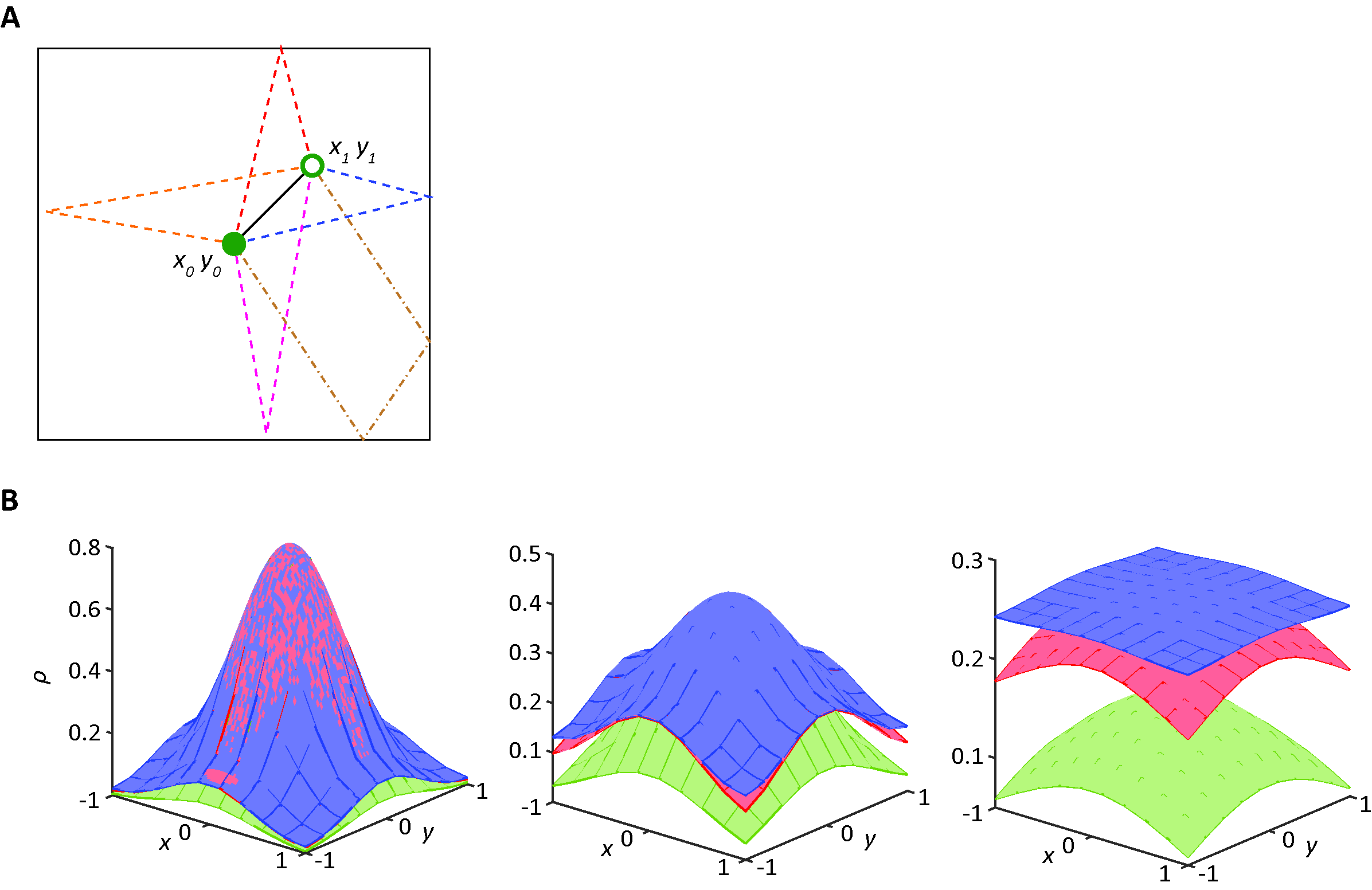

Supplement: S2 Fig — (A) Example of a rectangular billiard. We show all possible billiard trajectories that take the particle from p0 = (x0, y0) to pf = (x1, y1) after 0 (solid line) and 1 bounce (dashed lines), and we show one trajectory with 2 bounces (dash-dot lines). The solution of the diffusion equation in this scenario can be computed by adding all the “boundary-less” densities accounting for the different lengths of the billiard trajectories that take the particle from p0 to pf. (B) Comparison of the density ρ for a square of sides 2, with D = 2, ρ(x, y, 0) = δ2(x, y), and Neumann boundary conditions. In all figures the blue surface corresponds to the analytical solution, the green surface indicates the density without accounting for any bounce, and the red surface shows the density accounting for one bounce. Left for t = 0.01, center for t = 0.1, and right for t = 0.25. Notice that, as expected, the more bounces, the better the approximation. (TIF) [file pcbi.1011093.s005.tif]

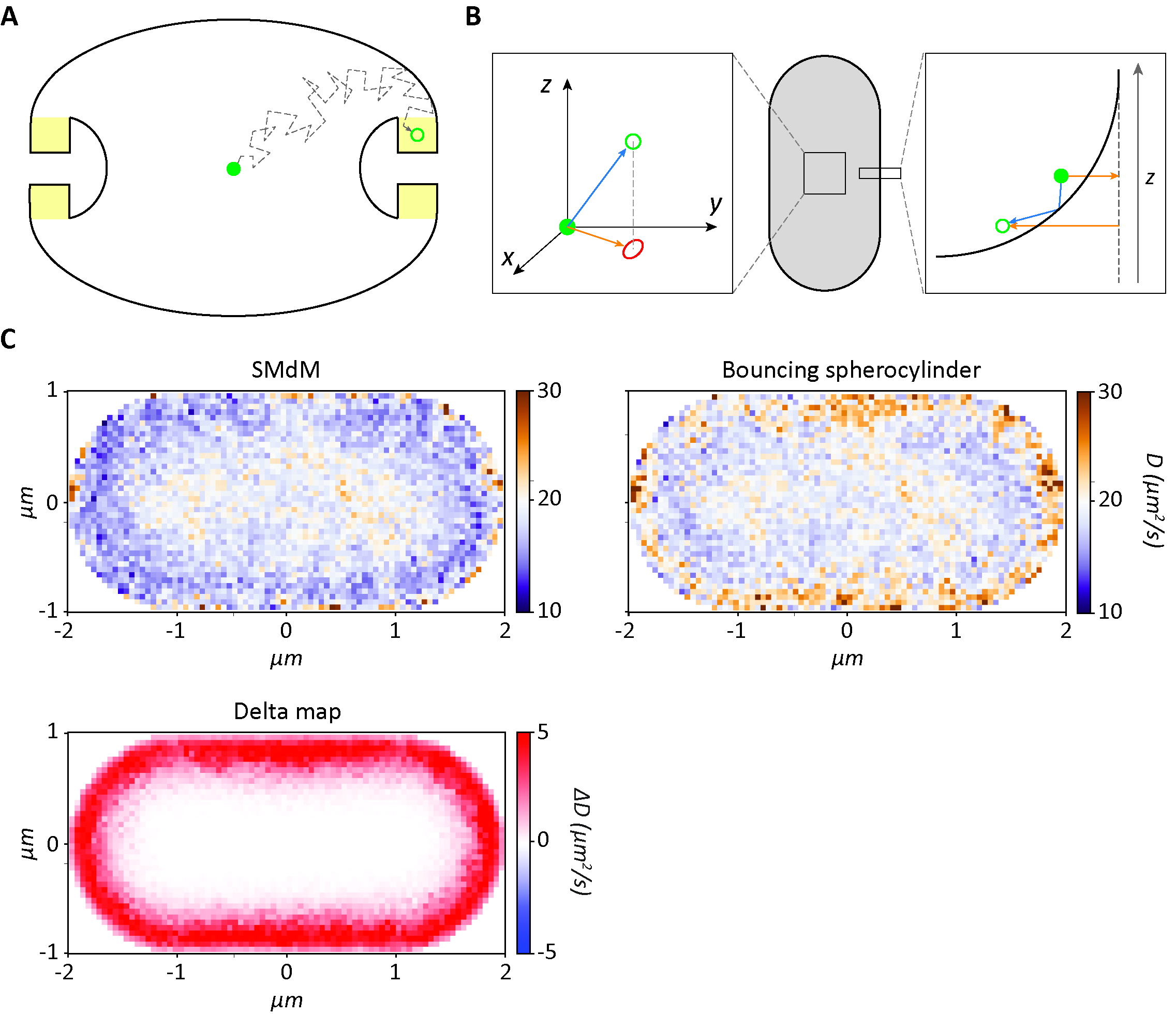

Supplement: S3 Fig — (A) Schematic of Penrose unilluminable room. A ray (vector) starting from the center of the room can never reach the regions colored in yellow, regardless of the number of bounces against the perimeter. A particle moving by random motion (dashed line) can reach any region in the room. (B) Limitations of using a 2D model to describe a 3D motion. Left panel: the motion of a particle moving in 3D space (blue arrow) is projected on a 2D surface (orange arrow). The observed distance is shorter than the actual travelled distance, leading to an underestimation of the diffusion coefficient. Right panel: the effect of the overestimation of the billiard perimeter. By observing the projection of a 3D spherocylinder in 2D, we use as billiard’s perimeter its largest xy projection. When displacements are binned in a pixel close to the boundaries, the bouncing of particles will likely occur against a different section of the spherocylinder, where the circumference of the billiard is smaller. In this way, the calculated bouncing path (orange arrow) overestimates the actual path (blue arrow), leading to an overestimation of the diffusion coefficient near the boundaries. (C) Diffusion maps of a spherocylinder obtained by analyzing a Smoldyn simulation created with an input diffusion coefficient of 20 μm2/s. Maps are obtained via SMdM analysis (left) and via mathematical method analysis (right). The difference between the mathematical map and the SMdM map is depicted in the bottom panel. (TIF) [file pcbi.1011093.s006.tif]

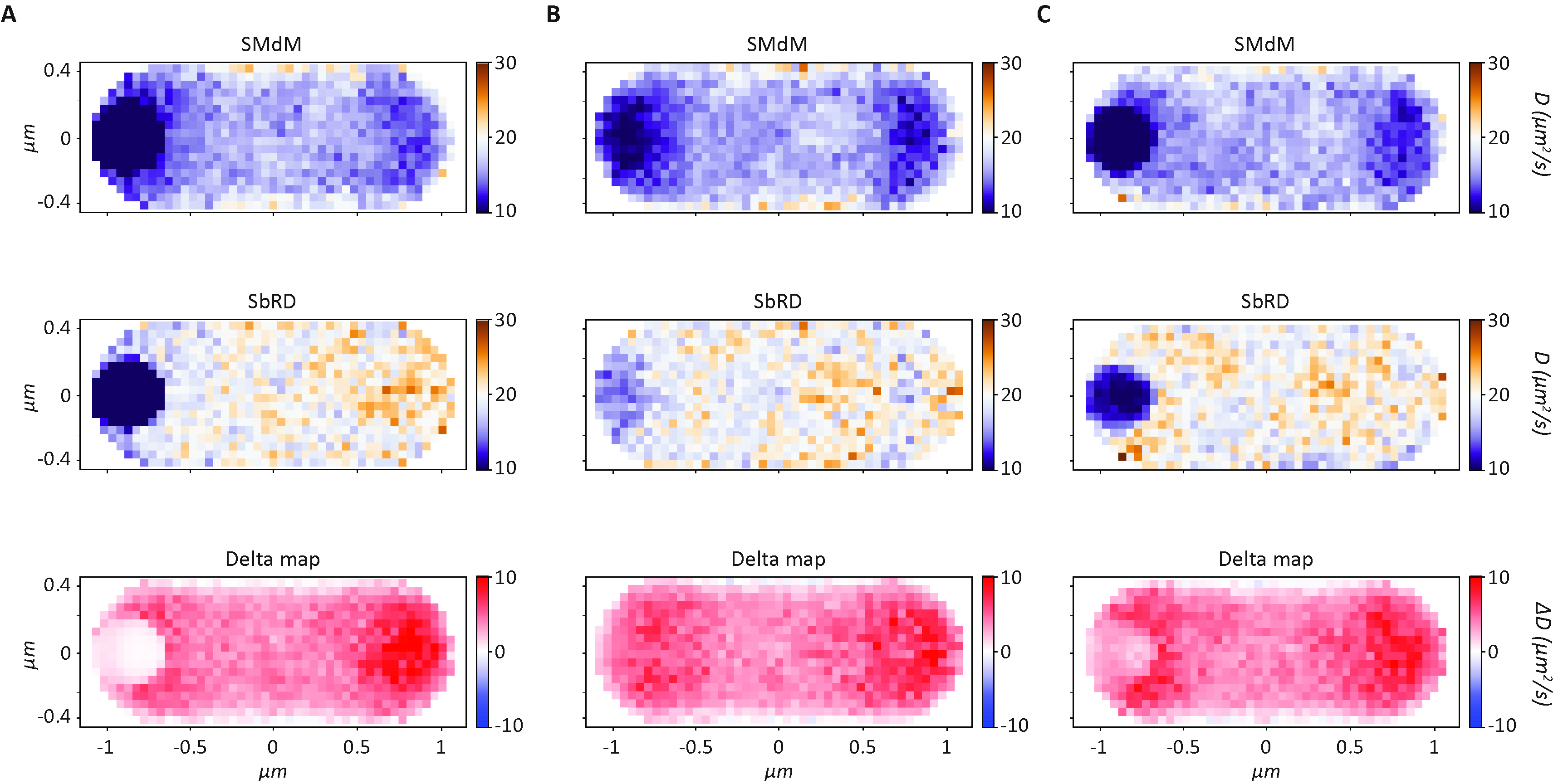

Supplement: S4 Fig — We simulated multiple component diffusion in a spherocylinder (using Smoldyn) with an input diffusion coefficient of 20 μm2/s for the first component. The second component was simulated with different dynamics. (A) Aggregation is simulated by defining a spherical region in the cell pole with input diffusion coefficient of 2 μm2/s. Particles diffusing outside of the aggregation region could not enter the sphere, and vice versa particles confined in the aggregation region could not diffuse outside of the sphere (B) Interaction with a cellular component located at the cell pole is simulated by defining a spherical region in the cell pole, in which particles change their diffusion coefficient from 20 μm2/s to 16 μm2/s upon entering the region and retrieve their original diffusion coefficient (20 μm2/s) after exiting the region. (C) Diffusion of two different species is simulated by defining a spherical region at the cell pole in which a species diffuses with D is 10 μm2/s, while the other species freely diffuse in the whole spherocylinder, including the spherical region, with a diffusion coefficient of 20 μm2/s. SbRD allows in all cases to retrieve information about the genuine diffusion coefficient in the cell and near the boundaries, without masking the effect of aggregation, interaction or multiple diffusing species. An advanced method using SbRD together with recursive recognition of slower or faster diffusing regions could allow detecting anomalies in cells. (TIF) [file pcbi.1011093.s007.tif]

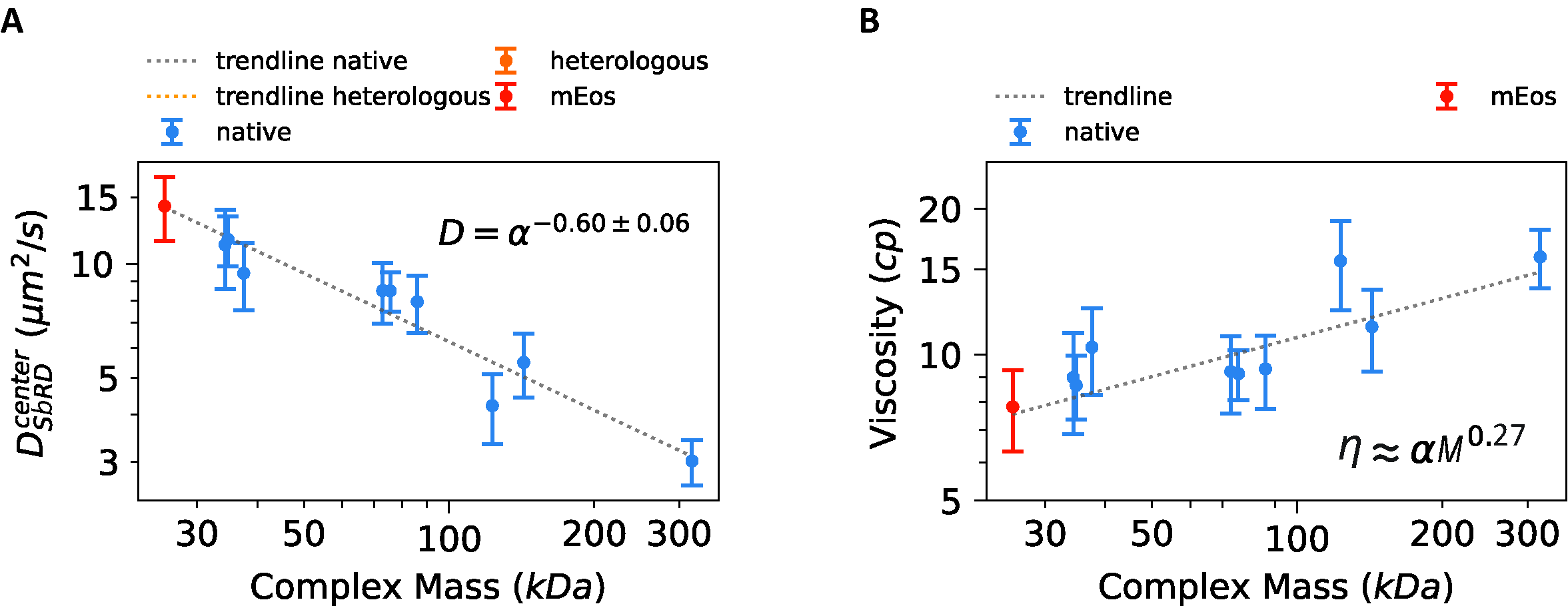

Supplement: S5 Fig — (A) Dependence of the Dcenter measured with SbRD on the complex mass of a set of cytoplasmic proteins [14] (S1 Table). Native proteins are indicated in blue, mEos3.2 is indicated in red. The gray trendline is obtained by calculating the dependence of the diffusion coefficient on the complex mass. (B) Intracellular perceived viscosity as a function of the molecular weight of protein complexes. The trendline is obtained by fitting the formula η = αM0.27, obtained by the difference between the Einstein-Stokes equation and our model for the relationship between diffusion and complex mass [14]. (TIF) [file pcbi.1011093.s008.tif]

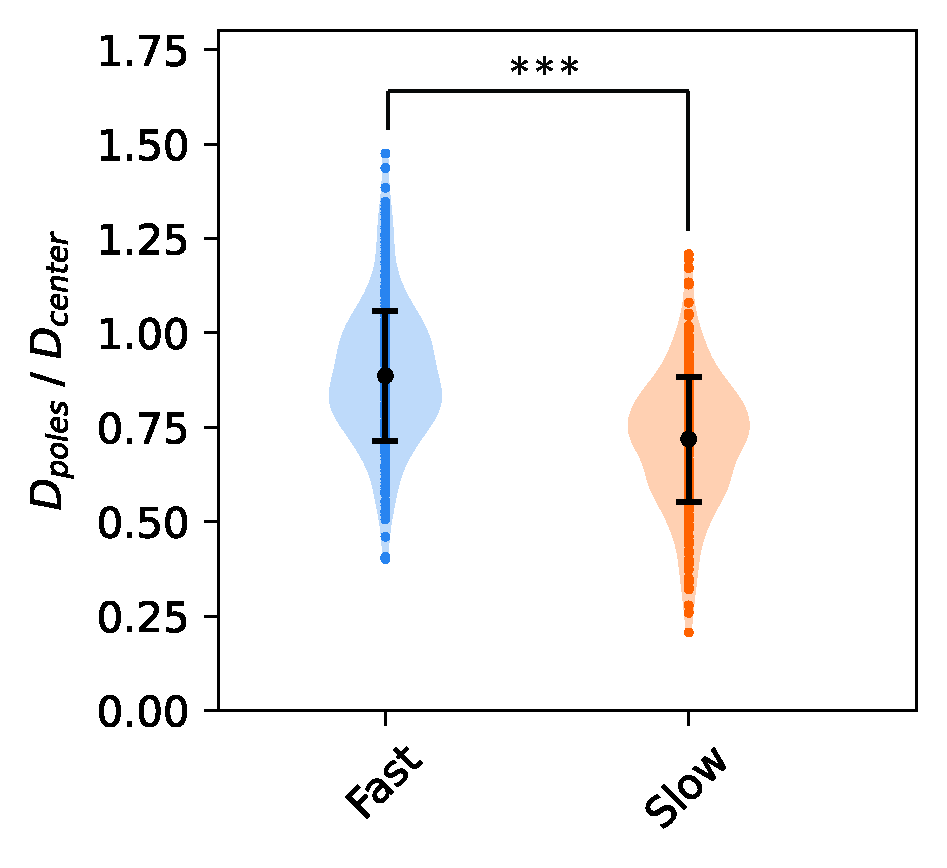

Supplement: S6 Fig — A previously acquired dataset [14] was re-analyzed with SbRD. The ratios between the diffusion values obtained at the cell poles and at the cell center were labeled as faster pole and slower pole for each cell. All faster poles were clustered together, and their value was compared with the clustered slower poles. The faster cell pole cluster has a value of 0.89 ± 0.17, while the slower cell pole cluster has a value of 0.72 ± 0.17. The difference in values between the two clusters is significant, with a p-value << 0.01, calculated from a Mann-Whitney U rank test for non-normally distributed samples. (TIF) [file pcbi.1011093.s009.tif]

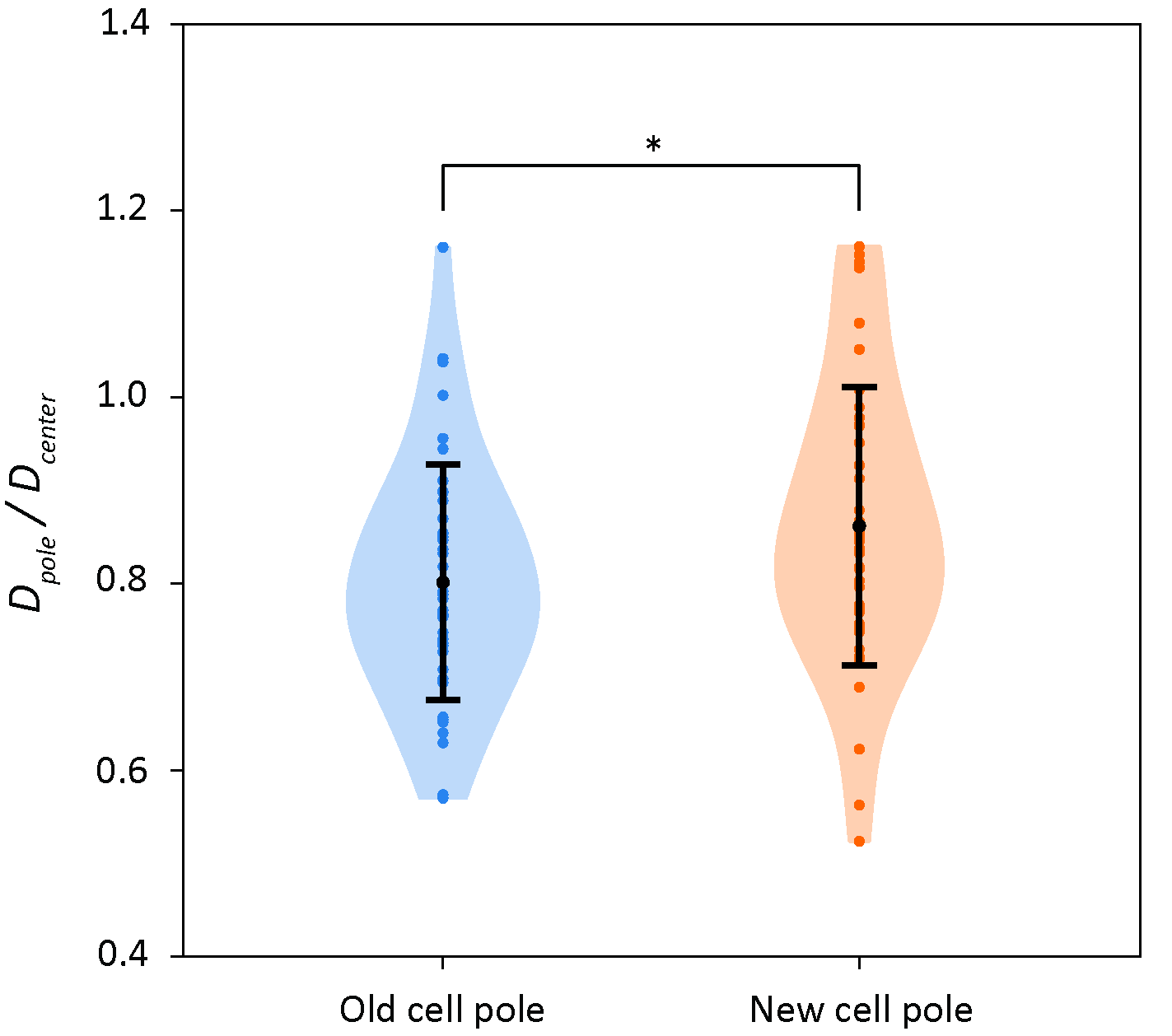

Supplement: S7 Fig — The acquired dataset of dividing cells was analyzed with SbRD. The presented data are averages of the whole dataset. The average of the ratios between the diffusion at the old cell pole and the diffusion at the cell center was compared with the average of the ratios between the diffusion at the new cell pole and at the diffusion at the cell center. The old cell pole cluster has a value of 0.80 ± 0.13, while the new cell pole cluster has a value of 0.86 ± 0.15. The difference in values between the two clusters is significant, with a p-value < 0.05, calculated from a Mann-Whitney U rank test for non-normally distributed samples. (TIF) [file pcbi.1011093.s010.tif]
