# Supplementary material for: Simulation-based Reconstructed Diffusion unveils the effect of aging on protein diffusion in Escherichia coli
Source: PLoS Comput Biol. 2023 Sep 11;19(9):e1011093. doi: 10.1371/journal.pcbi.1011093 (PMC10513214; doi:10.1371/journal.pcbi.1011093)
Supplement: S1 Table — The cell numbers represent single, non-dividing cells without visible aggregation of the expressed protein. The columns show the target protein, number of analyzed cells, abundance, loneliness, molecular weight, oligomeric state (1—monomer, 2—homodimer, 4—homotetramer), and complex mass. The complex mass is the sum of the molecular weight of the monomeric protein plus mEos3.2 and multiplied by the oligomeric state number. The mean and SD of Dapp center are shown in the last two columns. The UniProt ID is reported for all proteins, except for mEos3.2, for which the Fpbase ID is given. (PDF) [file pcbi.1011093.s011.pdf]

Supporting Table

Construct ID	UNIPROT ID	Protein name	Number of cells	Abundance (copies/cell)	Loneliness	MW (kDa)	Oligomeric state	complex mass (kDa)	D_{app}^{center}	
									mean	standard deviation
1	VUXFR*	mEos3.2	30	--	--	25.7	1	25.7	11.4	1.6
3	P00934	ThrC	31	11109	0.350	47.1	1	72.8	7.8	1.1
8	90AC62	GrxC	26	6170	89.400	9.1	1	34.8	10.3	1.3
9	P05793	IlvC	22	29065	36.200	54.0	4	318.9	2.9	0.5
11	P08997	AceB	28	8308	10.400	60.2	1	85.9	6.9	1.1
12	P0A6A8	AcpP	38	28863	0.120	8.6	1	34.3	9.6	1.6
13	P0ACC3	ErpA	22	3460	0.100	12.1	2	75.5	7.8	1.1
15	P0AA25	TrxA	33	18242	0.025	11.8	1	37.5	8.7	1.8
16	P07813	LeuS	20	1505	0.005	97.2	1	122.9	4.1	0.8
19	P08200	Icd	23	24591	1.020	45.7	2	142.8	5.0	0.9
15_hvo	A0A558GCJ2	TrxA2_hvo	23	--	--	12.1	1	37.8	8.1	1.2
15_lla	A0A089XQE8	TrxA_lla	24	--	--	11.7	1	37.4	6.6	1.3

Supporting Table 1. Dataset from (14). The cell numbers represent single, non-dividing cells without visible aggregation of the expressed protein. The columns show the target protein, number of analyzed cells, abundance, loneliness, molecular weight, oligomeric state (1 - monomer, 2 - homodimer, 4 - homotetramer)', and complex mass. The complex mass is the sum of the molecular weight of the monomeric protein plus mEos3.2 and multiplied by the oligomeric state number. The mean and SD of D_{app}^{center} are shown in the last two columns. The UniProt ID is reported for all proteins, except for mEos3.2, for which the Fpbase ID is given.
